# Cells of all trades: On the importance of spatial positioning of senescent cells in development, healing and aging

**DOI:** 10.1002/1873-3468.70037

**Published:** 2025-03-29

**Authors:** Helene Dworak, Tomaz Rozmaric, Johannes Grillari, Mikolaj Ogrodnik

**Affiliations:** 1 Ludwig Boltzmann Institute for Traumatology. The Research Center in cooperation with AUVA, Vienna, Austria; 2 Austrian Cluster for Tissue Regeneration, Vienna, Austria; 3 Institute of Molecular Biotechnology, BOKU University, Vienna, Muthgasse 18, Vienna, Austria

## Abstract

Biological processes are often spatially regulated, ensuring molecular and cellular events occur in their most strategically advantageous locations. Cellular senescence, marked by cell cycle arrest and hypersecretion, is recognized as an important part of physiological processes like development and healing, but it also contributes to aging and disease. However, the spatial distribution of senescent cells and its physiological and pathological impact remain unclear. Here we compile evidence on senescent cell localization in development, healing, and aging. We emphasize the significance of their spatial patterns and speculate on the effects of disrupted spatial positioning of senescence in relation to pathologies. To summarize the specific spatial functions of senescent cells, we propose to refer to them as 'barrier' and 'conductor' functions. The 'barrier' function of senescent cells, due to their altered morphology and apoptosis resistance, separates tissues and builds a border between two environments. The function as a 'conductor', with the secretion of signalling factors, influences the surrounding area and stimulates migration, differentiation, or proliferation, among other processes. Overall, this review explores the spatial patterning of cellular senescence in biological processes, highlighting its dual roles as 'barrier' and 'conductor' functions, and examines the implications of senescent cell distribution in development, healing, aging, and disease.

## Introduction

Biological processes are controlled in a highly specific spatial manner, i.e. they are restricted to an area where their function is the most needed and/or the most effective. The most obvious examples include separations between highly specialized cells of organs and sub-organ structures, like hepatocytes are present only in the liver, cardiomyocytes only in the heart etc. However, cells can exhibit different phenotypes and execute different processes not only due to differences in their identity, but also due to the spatially-restricted range of physiological or pathological stimuli. For example, epidermal cells acquire mesenchymal properties upon injury enabling re-epithelialization and wound closure. This process that is spatially limited to an area around the injury site [1]. Similarly, developing organs like the heart have specific, spatially restricted cues that lead to distinct cell lineages forming the organ [2], [3]. Thus, spatially-restricted cellular activities and phenotypes are the result of cellular identity or external stimuli. The majority of what we know about spatially-restricted phenotypes comes from the fields of developmental sciences, regeneration, and wound healing where the correct spatial patterning is essential for proper growth or re-growth of biological structures. The science of aging, biogerontology, has yet to address its core questions related to spatial biology, such as whether aging occurs at a different pace between organs, types of cells or sub-regions. In this regard, it is possible to make some predictions regarding the significance of spatial aspects of aging based on the evidence collected on the process connecting the aging process with the sciences of development and wound healing, cellular senescence.

Cellular senescence is a phenotype of pro-secretory cell cycle arrest occurring in various types of cells and across organs. A description of senescent cells that relies on a single biomarker is inadequate; rather, a more comprehensive approach is necessary, taking into account the various attributes associated with this phenomenon [4]. Senescent cells are distinguished by elevated expression of cell cycle arrest proteins p16^INK4A^ or p21^CIP1/WAF1^ (from this point forward, p16 and p21, respectively), as well as elevated expression of secretory molecules referred to as senescence-associated secretory phenotype (SASP). Furthermore, morphological changes including soma enlargement, resistance to apoptosis and altered metabolism are notable, which is evident in the accumulation of lipid droplets (termed “accumulation of lipids in senescence” (ALISE)) and impaired lysosomal activity, which can be visualized as senescence-associated-beta-galactosidase (SA-β-gal) [4]. It has been demonstrated on numerous occasions that that the phenomenon of cellular senescence is a significant contributing factor to age-related diseases [5], [6]. In the field of physiology, the presence of senescent cells has been demonstrated to influence the processes of healing and development. The inhibition or elimination of these cells has been shown to result in a reduction in the rate of regrowth and incomplete development of specific biological structures, respectively [7]. It is unclear whether there is a specific spatial pattern behind the induction of senescence in healthy and diseased states, or whether the spatial positioning of senescent cells impacts their influence on animal physiology.

The majority of existing research is focused on the assessment of the presence or absence of senescent cells in tissues using bulk quantitative approaches, such as polymerase chain reaction (RT-qPCR) or Western blot (WB). These methods, however, fail to consider the spatial distribution of senescent cells. An investigation into the spatial localization of senescent cells may elucidate the factors that trigger senescence and the relationship between their spatial arrangement and function. This review article presents a summary of the evidence on the spatial distribution of senescent cells and their impact on physiology and pathology. Additionally, it describes the potential causes of a specific pattern of spatial distribution of senescent cells in tissues. Finally, it offers speculation on the importance of spatial positioning of senescent cells and the implications of disruptions to this process in conditions such as aging.

### Evidence on the importance of spatial positioning of senescent cells during development

During the development, the establishment of cellular phenotypes is contingent upon the activation of soluble factors, among other factors. The diffusion capacity and other physical limitations establish a specific range for phenotype determination, as these signals are spatially restricted [8]. The spatial regulation of development is primarily accomplished in a cell-autonomous and in a paracrine manner.

While a coordinated regulation of cell phenotypes, including proliferation, migration and differentiation, plays a pivotal role in embryonic development, mounting evidence suggests that cellular senescence is another crucial regulator. Senescent cells are present in specific anatomical structures of murine embryos and exhibit distinct patterning in locations, including the apical ectodermal ridge (AER) of the limb, the closing neural tubes, the tip of the tail, the hindbrain roof plate, the mesonephros, and the endolymphatic sac, among others [9], [10], [11].

Senescent cells identified in development are distinguished by SA-β-gal staining, as well as other characteristics of senescence, including cell cycle arrest mediated by p21 or p27, with the notable exception of p16 [10], [11]. It is noteworthy that these cells exhibit no indications of DNA damage [10], which is frequently utilized as a marker for senescence in the contexts such as ageing [12]. They are negative for Ki67 staining or BrdU incorporation, indicative of proliferation arrest and display alterations in heterochromatin markers like H3K9me3 [10], [11], [13], [14].

**Figure 1:**
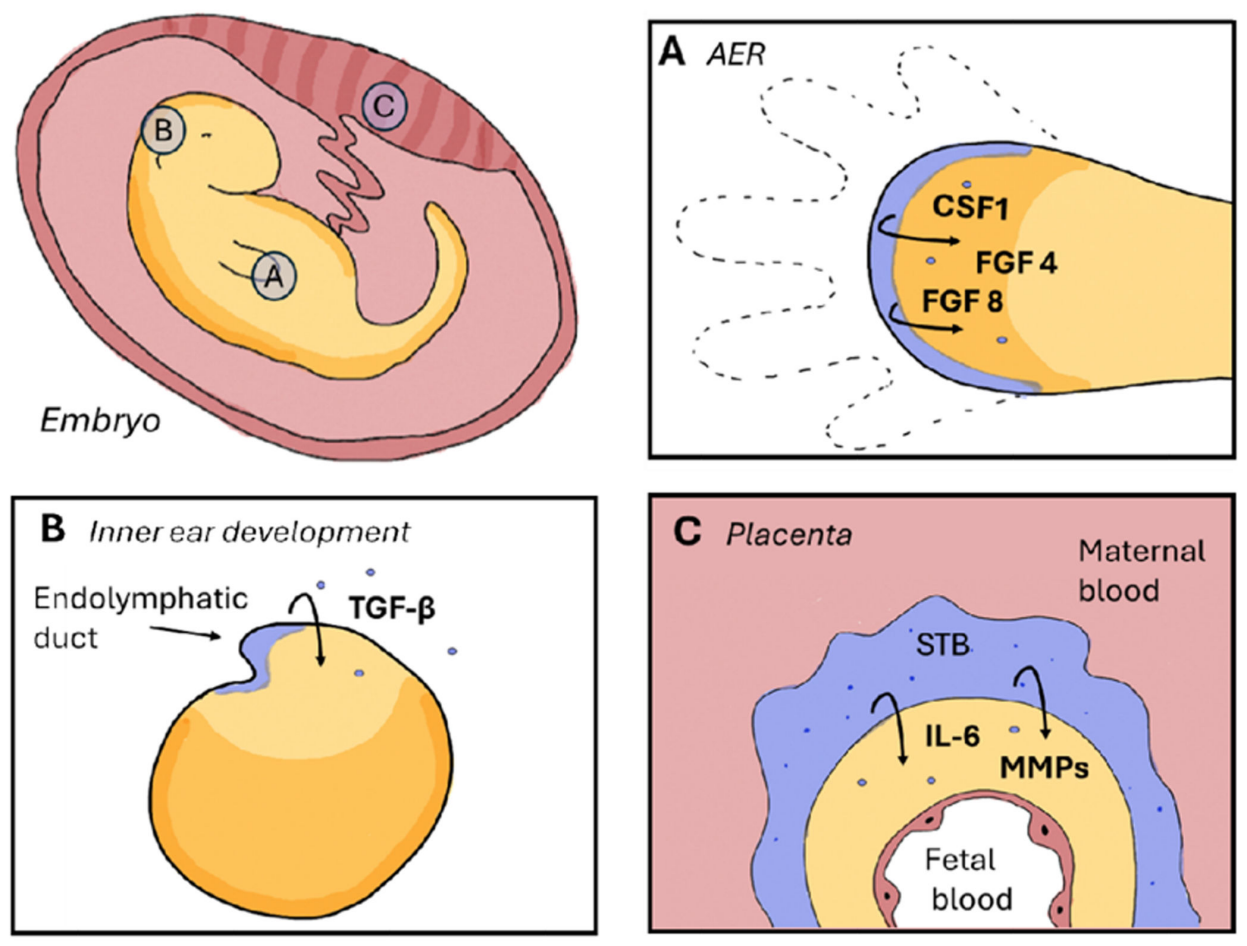
Spatial positioning of senescent cells in development. (A) During limb development a thin layer of senescent cells are found at the edge of the apical ectodermal ridge (AER) which stimulates due to secretion of CSF1 and FGF the growth of the underlying tissue. (B) During development of the inner ear senescent cells appear at the edge of the closing pores of the otocyst at the endolymphatic duct (Ed) in an TGF-β dependent mechanisms, which is essential for correct morphogenesis. (C) During embryogenesis the syncytiotrophoblast (STB), a multinucleated, fundamental structure between mother and foetus, forms and displays senescent features, like secretion of MMPs and IL-6, which are viable for the full function of the placenta. Senescent cells indicated in blue.

Senescent cells have been observed in major signalling centres at the edges of developing tissue, such as the AER (Fig. 1A) and among the epithelial cells in the roof plate of the hindbrain [10]. The AER is a specialized ectodermal region that demarcates the dorsoventral boundary of the limb bud [15] and serves as a crucial signalling hub that governs both the expansion and patterning of the limb [16]. The proliferation of the underlying mesenchyme is influenced by the AER via the secretion of growth factors including fibroblast growth factor (FGF) 4 and 8 [17]. The *in situ* hybridization of FGF8 identifies the AER as a thin layer at the tip of the developing limb at embryonic day (E) 11. This distinctive pattern of a narrow band at the edge of the developing limb precisely aligns with the pattern observed in SA-β-gal staining [10]. Gene-expression profiling of the AER reveals an overlap with senescence and critical mediators like p21, and growth factors like FGF, but also insulin-like growth factor-binding protein 5 (IGFBP5) and the macrophage recruiting factors colony-stimulating factor 1 (CSF1) [10]. Fate mapping studies have demonstrated that the AER initially forms as a narrow band at the distal tip of the developing limb bud around embryonic day E11 [18]. As development progresses, cells from the AER accumulate in the interdigital spaces by E14.5. Around E17.5, they undergo macrophage-mediated clearance, which marks the end of the AER’s functional role. It is noteworthy that this structure is absent in the dorsal ectoderm and remains localized to the ventral region, where it patterns the limb tip before its eventual clearance before birth [18]. Following the same pattern at E14.5, also the majority of senescent cells are located in the interdigital mesenchyme and are prompted to undergo clearance by macrophages [10].

In p21-deficient mice, a notable decline in cell proliferation within the mesenchymal tissue situated directly beneath the AER was evident [10]. This reduction indicates that the capacity of the AER to stimulate growth in the underlying tissue is impaired in the absence of p21 – and thus presumably in the absence of senescent cells. The absence of p21 results in a reduction in the expression levels of essential growth factors, namely FGF4 and FGF8, which are key signaling molecules involved in the promotion of cellular proliferation in the mesenchyme. Moreover, p21 plays a protective role by preventing apoptosis within the AER. In the absence of this senescence-mediated protection, AER cells are more susceptible to undergoing apoptosis, which in turn disrupts the proliferation of the underlying mesenchymal cells. This series of events ultimately impedes the typical growth and patterning of the developing limb [10].

A follow-up study on the characterization of senescent cells in development raised a point of controversy on the stability of their cell cycle arrest [19]. Fate mapping of senescent cells demonstrated that they are not exclusively cleared by macrophages, but a subset can re-enter the cell cycle. Specifically, from the thin layer of SA-β-gal- and p21-positive cells in the AER, some cells were shown to resume proliferation, thereby contributing to tissue growth postnatally, suggesting a previously unappreciated plasticity in the fate of senescent cells *in vivo* [19]. It remains unclear whether these properties are consistent across senescent cells in various contexts; therefore, fate mapping of cellular senescence is necessary to elucidate such unexpected phenomena.

The spatial distribution of activated extracellular signal-regulated kinase (ERK) correlates with critical developmental processes, such as cell fate determination and tissue patterning [20], [21]. The precise localization of ERK signaling is essential for the proper specification of the anterior-posterior axis and the differentiation of mesodermal tissues [22], [23]. In the context of senescence, ERK signalling represents an essential mediator of the cell cycle initiation and the induction of the SASP [24], [25]. Storer et al investigated whether the suppression of ERK signalling would result in a notable reduction in senescence in the AER [10]. Indeed, the inhibition of ERK signalling by injecting pregnant female mice at E11.5 with the MEK inhibitor U0126 led to a decrease in senescence in the AER. Consequently, this resulted to abnormalities in the developing limb, indicating that ERK-driven senescence is mediating the correct patterning of tissue at distinct stages of development [10].

Another process in embryogenesis is the development of the inner ear, which is a spatially-organized process occurring between E8.5 and E17 [26]. The precise patterning and development of the structure preceding the formation of the inner ear, the otocyst, plays a pivotal role in the development of hearing and balance in vertebrates [27] (Fig. 1B). Senescent cells manifest in a distinctive pattern, initially encircling the otic vesicles and subsequently localizing to the otocyst at the endolymphatic duct [27]. The highest concentration of senescent cells is observed at the edges of the pores, which eventually will close to form the otocyst duct [27]. At E14.5 a very thin layer of p21- and SA-β-gal-positive endothelial cells is detected. These senescent cells modulate the endolymphatic duct morphogenesis by releasing signalling molecules, including transforming growth factor beta (TGF-β) [27]. This superfamily of pleiotropic cytokines is evolutionarily conserved and contributes to a variety of cellular processes, including differentiation, proliferation, apoptosis and migration in a context-dependent manner [28]. The TGF-β signalling pathway is associated with the process of aging and cellular senescence, given its role in the inhibition of cancer growth. However, its chronic upregulation is linked to the development of chronic inflammation and fibrosis [29]. The dependency on the TGF-β pathway for the proper morphogenesis of the inner ear was validated through the administration of TGF-β pathway inhibitor during mouse pregnancy [14]. In the same study, the direct involvement of senescence in the development of the inner ear was further demonstrated with p21-KO mice. Accordingly, embryos of p21-KO mice exhibited abnormal endolymphatic sac folding. However, these abnormalities were undetectable in adults, leading to the conclusion that a macrophage-dependent, post-development remodelling process of the inner ear must be operative and be independent from senescence [11].

So-far, most of the abovementioned examples indicate that senescent cells exert an influence on development through a specific combination of SASP factors, including growth factors such as TGF-β and FGFs in a non-cell autonomous way.

However, at the interface between mother and foetus, the placenta, spatial positioning of senescent cells contributes to development in a cell-autonomous manner. At the core of the placental connection between mother and the foetus is the syncytiotrophoblast (STB), an extraordinary biological structure that arises primarily from cell fusion and is comprised of a single interconnected cytoplasm that houses tens of billions of nuclei [30]. It has been demonstrated that the fusion of cells is a prerequisite for the onset of cellular senescence, which is a crucial process for the generation and maintenance of placental STB [31], [32]. Markers of cellular senescence, including elevated levels of p16, p21 and elevated activity of SA-β-gal, are expressed in human placental STB cells at the interface between maternal and foetal tissues (Fig. 1C). These facilitate the stable cell-cycle arrest of the STB, which is required as uncontrolled cell division would have disastrous consequences for such a large and complex tissue [31].

Within the STB, the key features of senescent cells, such as resistance to apoptosis and a flat morphology, facilitate the formation of a barrier system between the mother and foetus, which is important for the functional transfer of nutrients [33], [34]. In embryogenic development, the precise spatial coordination of the deposition and remodeling of the extracellular matrix (ECM), comprising collagens, glycosaminoglycans, and proteoglycans, plays a pivotal role in morphogenesis [35]. During the first trimester, the secretion of matrix metalloproteinases (MMPs) by the STB are vital for the very early placentation, but in later stages, it also contributes to the remodelling of the cervical ECM, which is necessary for the preparation of the birth canal [36].

In addition to these cell-autonomous functions, the production of cytokines by senescent cells, including IL-6, MMP2 and MMP9, and their paracrine attraction of natural killer (NK) cells are crucial for maintaining optimal placental function [32]. By employing non-invasive *in utero* monitoring of placental function and vascularisation by dynamic contrast-enhanced magnetic resonance imaging (DCE-MRI), abnormalities were discovered in senescence-attenuated (p21-KO) mice when compared to WT placentas [32]. In humans, intrauterine growth restriction (IUGR) represents one of the most prevalent complications encountered during pregnancy. Abnormalities in placental growth, structure, and function are closely linked to this phenomenon, making it a significant contributor to fetal morbidity and mortality. In accordance with the murine experiments, the central senescence pathways are attenuated in IUGR placentas, and decreased levels of SASP factors are detected. This underscores the significance of senescence induction in the STB for optimal placenta function [32]. Furthermore, the vascularization of the placenta seems to be mediated by cellular senescence, as evidenced by the collapse of the placental vasculature from p21 KO mice [32].

While the aforementioned examples indicate that senescent cells are beneficial in the context of development, there is evidence that increasing their number beyond the programs of the development or inducing them ectopically (outside of places where they are induced by the developmental programs) might be detrimental. Valproic acid (VPA), a pharmaceutical agent utilized for the treatment of various medical conditions, including epilepsy, has been demonstrated to elevate the risk of congenital anomalies when administered during the gestational period. VPA-treated mouse embryos at E9.5 exhibit developmental abnormalities, including reduced brain size (~40%) and/or open neural tubes (~30%) [37], [38]. The assessment of senescence in whole-mount staining revealed robust SA-β-gal activity in the forebrain and hindbrain, particularly at the apical border of neuroepithelial cells in embryos treated with VPA. Thus, spatially abnormal induction of senescence during embryogenesis is therefore strongly associated with developmental defects [38].

Likewise, maternal diabetes represents a significant risk factor for the development of structural defects, including those affecting the neural tube [39]. In mice with streptozotocin-induced diabetes, neural tube defects in the foetus are identified and correlate strongly with premature and ectopic induction of cellular senescence [39]. As pregnancy nears its end, particularly in the post-term period, the foetus’s demand for oxygen and nutrients may exceed the capacity of the placenta to provide them [40]. This imbalance results in oxidative stress, which causes the increased levels of NOX4 and, consequently reactive oxygen species (ROS) in both post-term and pre-eclamptic placentae [40]. Even though senescent cells – STBs as outlined above - are part of the normal physiological development of the placenta, their accelerated accumulation due to oxidative stress is increased in post-term or pre-eclamptic placentae compared to placentae from women delivering at term [40]. *Ex vivo* placental studies conducted under normoxic or hypoxic conditions with reperfusion demonstrated a notable elevation of p21 and DNA damage at the edges, which was attributed to oxidative stress [41]. Increased oxidative stress can lead to placental dysfunction, which can result in stillbirth, reinforcing the importance of the temporal and spatial constraints associated with the process of aging [40], [42].

In summary, senescent cells are present in spatially restricted developmental structures, including the AER of the limb and the roof plate of the hindbrain. In these contexts, they modulate tissue patterning and cellular proliferation via paracrine signaling, with their timely clearance being essential for proper morphogenesis, such as in limb development and inner ear formation. In the placenta, senescence facilitates tissue remodeling and vascularization, underscoring its role in maintaining placental function. However, mis-timed or ectopic induction of cellular senescence can result in developmental abnormalities, emphasizing the importance of precise spatial and temporal patterning of senescent cells during development.

### Evidence on the importance of spatial positioning of senescent cells in healing

Wound healing is a highly orchestrated process that requires the coordinated interplay of multiple cell types to ensure successful tissue repair and restore the integrity of the skin. Following an injury, a sophisticated interplay of cellular signals determines the spatial distribution of a range of processes, including proliferation, apoptosis, migration, and ECM remodeling [43]. The spatial organization of cells is of great consequence in directing the healing process, as the distance of cells from the wound site plays a pivotal role in determining their function. For instance, keratinocytes in close proximity to the injury site are responsible for migration and re-epithelialization, whereas those situated at a greater distance from the wound are more proliferative [44]. During re-epithelialization, keratinocytes migrate from the wound edges toward the center, a process that is tightly regulated by spatial cues such as gradients of growth factors like epidermal growth factor (EGF) and TGF-β. These gradients guide not only keratinocytes but also endothelial cells and fibroblasts, which contribute to new blood vessel formation and connective tissue repair, respectively [45], [46]. Overall, injury-induced changes in post-translational modifications, gene expression, cytoskeletal organization, and other cellular processes that occur in response to spatial cues are of great importance for the distribution of tasks associated with wound closure and effective healing [47]. In addition to the processes of death and proliferation, another cell fate that is induced upon injury is senescence, as senescent cells are increasingly being recognized as a key factor in the process of in wound healing [48], [49]. Furthermore, other markers of senescence have been observed in these cells, particularly in those exhibiting high p21 expression. These markers include alterations in cell morphology and nuclear envelope structure, as evidenced by decreased LMNB1 levels, as well as changes in metabolic activity, characterized by an increase in lipid droplets, which can be detected through Perilipin 2 (PLIN2) staining [4], [50]. The senescent phenotype of p21-positive cells was additionally verified through the utilization of single-cell RNA sequencing (sc-RNA-seq) [50].

Senescent skin cells, like fibroblasts and keratinocytes, release SASP factors, which consist of various chemotactic signals attracting immune cells, including neutrophils, monocytes, NK, T and B cells, along with mast cells, to the injured area [49], [51]. The recruitment of immune cells to the site of injury facilitates the clearance of damaged tissue during the early stages of healing and the resolution of inflammation during the late stages [52], [53]. In addition to chemokines, the SASP includes inflammatory cytokines like IL-6 and IL-8, growth factors, and MMPs [54]. IL-6 and IL-8 play key roles in sustaining cellular communication and inflammatory signaling within tissues [55]. These cytokines are also essential for wound healing, as mice lacking IL-6 exhibit significantly delayed healing, with impaired re-epithelialization and healing times about three times longer [56]. Additionally, senescent cells play a pivotal role in cellular plasticity, especially through the secretion of SASP factors, including IL-6 [7]. They can induce de- or trans-differentiation in their neighboring cells, such as the transformation of fibroblasts into myofibroblast, a specialized form of fibroblasts [57]. These myofibroblasts, which typically accumulate in the wound’s center, exert contractile forces, which are essential for wound closure [43], [58], [59]. It has been demonstrated *in vitro* that the trans-differentiation of non-senescent fibroblasts into myofibroblasts is enhanced by the presence of senescent fibroblasts [60]. Moreover, secretion of IL-6 by keratinocytes stimulates differentiation into myofibroblasts at the edges of the wound [59], [61]. While it has not been demonstrated that these keratinocytes co-express markers of senescence, the expression of IL-6, particularly by keratinocytes in close proximity to the wound edge, is crucial for the formation of granulation tissue and the promotion of re-epithelialization [56], [62]. Finally, IL-6 plays a pivotal role as a mediator of cellular reprogramming [68]. A mouse model utilizing doxycycline-inducible transcription factors Oct4, Sox2, Klf4, and c-Myc (OSKM) has revealed that the reprogramming of neighboring cells is diminished when senescent cells are eliminated or when the level of IL-6 is reduced [68]. Overall, pluripotency and senescence are intertwined processes: cellular damage causes senescence, which in turn enables the induction of pluripotency in neighboring cells [69]. Conversely, the induction of pluripotency can cause cell damage and senescence in close proximity, both *in vivo* and *in vitro* [70].

The beneficial role of senescent cells for wound healing has been elucidated through the use of p16/p21 double knockout (DKO) mice, which demonstrate a delayed wound healing process in comparison to their WT counterparts [48]. Similarly, the p16-3MR mouse model allowing to identify and eliminate senescent cells by ganciclovir [48], show that selective eradication of p16-positive senescent cells (senolysis) resulted in a markedly prolonged wound healing process [48]. This study has also shown that senescent fibroblasts are predominantly situated in the vicinity of the granulation tissue adjacent to wound margins. These wound-associated senescent cells secrete a distinctive SASP factor, PDGF-AA, which accelerates wound closure by promoting myofibroblast differentiation, among other mechanisms [48]. Finally, in p16/p21 double knockout (DKO) mice, the administration of topical PDGF-AA, to compensate for the missing factor normally secreted by senescent cells, rescued the delayed wound healing. Taken together, these results emphasize the pivotal role of senescence and the SASP in the process of wound healing [48].

**Figure 2:**
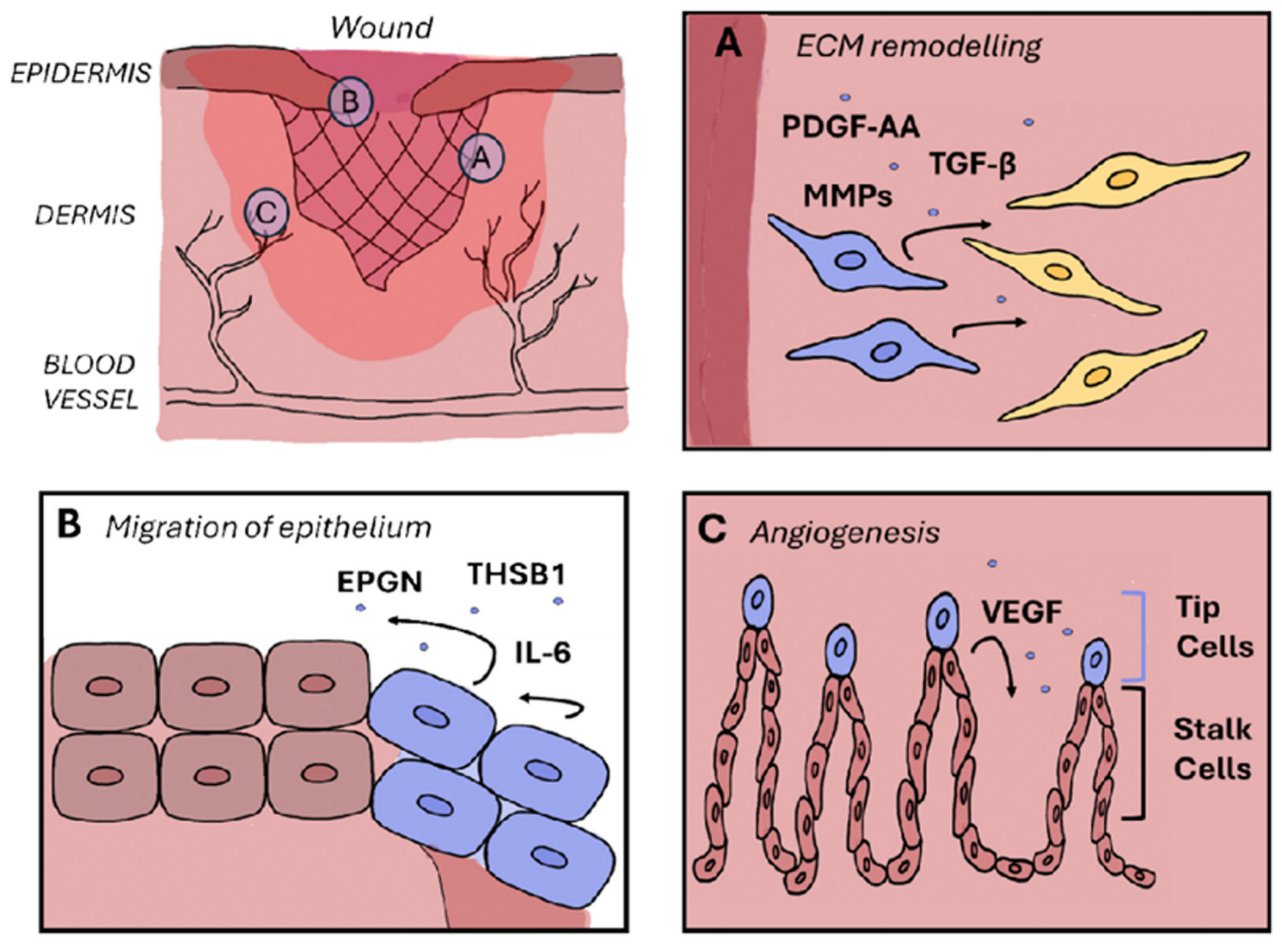
Spatial positioning of senescent cells during wound healing. (A) Fibroblasts at the edge of the wound become senescent and many SASP factors, like PDGF-AA, MMPs and TGF-β are essential for wound closure. (B) Migrating front of keratinocytes during wound healing have senescent features like expressing of p21 and trough the secretion of SASP factors like EPGN, THSB1 and IL-6 lead to the re-epithelisation of the wound. (C) During angiogenesis the tip cells arrest in their cell-cycle and lead the migrating and sprouting of new blood vessels. Senescent cells are indicated in blue.

Another essential aspect of wound healing is the re-deposition of the ECM, which requires careful regulation to prevent excessive fibrosis and scarring. ECM components such as collagen are secreted primarily by fibroblasts which migrate into the wound bed and lay the groundwork for new dermis. During specific phases of wound healing, senescence regulators such as p53, p21 and p16 enforce cell cycle arrest in fibroblasts and prevent cell proliferation [63]. The secretion of MMPs by senescent fibroblasts helps to remodel the ECM during wound healing and plays a critical role in limiting fibrosis [64]. The induction of cellular senescence results in a switch from ECM production to degradation in a cellular communication network factor 1 (CCN1)-dependent manner and restricts fibrosis [64]. The secretion of MMP2, MMP3 and MMP9 near the wound edge results in the degradation of the provisional matrix laid down during the early phases of healing, which allows for tissue maturation [64]. During healing, while initially proliferating, myofibroblasts that produce ECM are eventually cleared by the immune system, thus self-limiting fibrosis [57], [65], [66]. The localization of senescent cells to areas where ECM breakdown is needed ensures that matrix remodeling is spatially restricted to regions that require it, thereby limiting fibrosis and scarring (Fig. 2A). Furthermore, MMPs play a crucial role in wound healing beyond ECM degradation. It is essential for promoting cell migration, reorganizing the actin cytoskeleton, and facilitating elongation of cells at the wound edge, which is necessary for re-epithelialization of the basement membrane [67]. The spatial localization of senescent cells is of critical importance for these effects to be properly confined to specific regions of the wound, thereby limiting potential damage to surrounding tissues and preventing excessive scarring. While the aforementioned studies have demonstrated the role of senescence in paracrine signaling-induced ECM remodeling and cell plasticity, among other processes, relatively little attention has been given to the specific location of senescent cells in relation to wounds.

To provide an accurate spatial description of the injury response, we have recently developed a method that allows for the precise visualization of the skin volume around the injury that is actively involved in the healing process [68]. The cells within this defined zone surrounding the injury site undergo phosphorylation of the ribosomal protein S6 (p-rpS6), thus its name “p-rpS6-zone”. The p-rpS6-zone emerges within minutes of wounding and persists throughout the entire healing process. At approximately the same time, another research group demonstrated the induction of p-rpS6 during limb amputation in axolotls, establishing its crucial role in the regenerative process [69]. During the course of our research, we observed the presence of cells positive for p21 and telomere-associated foci (TAF) selectively within the p-rpS6-zone and never outside of it [68]. In another study, we further confirmed that p21-positive cells at the periphery of injured skin, within the p-rpS6-zone, exhibited characteristics of cellular senescence, including reduced levels of LMNB1, compromised proliferative capacity, and increased lipid droplets [50]. The spatial confinement of senescent cells within the p-rpS6-zone provides compelling evidence of their role in orchestrating the wound healing response.

Another fundamental aspect of the healing process is collective cell migration, which is made possible by the coordinated movement between leader and follower cells [70]. This process is dependent on the presence of chemical or mechanical guidance cues, which ensure directional migration. We have discovered that the front of the epidermal tongue, which is responsible for driving keratinocyte migration to facilitate wound closure, exhibits an upregulation of senescence markers but also SASP factors, including epigen (EPGN) and thrombospondin 1 (THBS1) [50] (Fig. 2B). The data consistently indicate that a reduction in the number of senescent cells and the subsequent lowering level of EPGN expression levels result in a compromised migration capacity of cells. This evidence suggests that senescent cells play an essential role in the migration of cells in wounds [50]. While outside of wound healing research, a study by Kim et al., has reached similar conclusions in the context of thyroid cancer, demonstrating that senescent cells lead the collective migration of cancer cells [71]. Senescent cells present in the invasive border at the front of papillary thyroid carcinoma induce migration through the upregulation of SASP factors, including MMPs and CXCL12/CXCR4 signaling, which guides cells along the gradient [71]. Moreover, the suppression of cell proliferation via p21 and the enhanced secretion of cytokines, such as IL-1α, IL-6 and IL-8, leads to collective cell migration also in breast epithelial cells [72]. In the context of healing, the presence of non-proliferating, p21-positive endothelial cells was observed at the front of migrating and growing blood vessels [73]. During angiogenesis these tip cells of the angiogenic front display exceedingly high levels of ERK activity and vascular endothelial growth factor (VEGF), which induces p21, cell-cycle arrest and migration [73], [74]. These senescent cells lead the migrating front, which is followed by proliferative stalk cells (Fig. 2C). In sum, these findings illustrate that senescent cells *in vivo* are spearheading the migration process, and their spatial positioning at the forefront of an injury site is crucial for the optimal execution of this function [73].

Although seemingly contradictory, the wounding-associated increase in proliferation is also associated with cellular senescence. Our own findings [50] and those of other researchers [52], [75] demonstrated that in regions situated more distally from the wound site, where senescence is not induced, there is a pronounced increase in proliferation, as evidenced by EdU incorporation. This pattern illustrates the differential spatial organization of cellular fates and mechanisms. The more distal areas are engaged in active cell proliferation, which is necessary for the generation of new cells to facilitate wound closure. In contrast, the proximal regions are primarily involved in inflammatory responses and the secretion of needed cytokines, which are essential for the initiation and maintenance of the inflammatory response. The unique localization of senescent cells near the wound site, in contrast to their absence in other regions, underscores the functional role of these cells in orchestrating the initial stages of wound healing.

Effective wound healing hinges not only on spatial positioning, but also on temporal coordination of cellular phenotypes for effective healing. Our recent research has revealed that the induction of cells positive for a wide range of senescence markers can occur as early as 1.5 h after wounding [50]. Our findings indicate that this rapid-onset induction of senescence is transcription-independent and dependent on the pre-existing transcripts of *Ckdn1a*. This again highlights the importance of senescence in specific conditions, as skin cells already have the *Cdkn1a* transcripts and thereby are ready to immediately response to external stimuli by undergoing senescence [50].

The complexity of senescence in wounds is reflected in the paradox that disrupting senescence signaling near the wound significantly delays the healing process [48], however the elimination of these senescent cells by immune cells in later stages of healing is important to ensure proper wound closure and to avoid chronic inflammation [52]. Similarly, drugs specifically designed to eliminate senescent cells, senolytics (which have been extensively reviewed elsewhere: [76], [77]) have been shown to accelerate healing of skin injuries, e.g. as inflicted by irradiation [78], [79]. Our recent study might resolve this dilemma by showing that the elimination of senescent cells specifically very early after injury disrupts the healing process of acute wounds [50].

In summary, senescent cells are of great importance in the process of wound healing, particularly due to their secretion of high levels of MMPs, PDGF-AA, EPGN and other SASP factors, which contribute to tissue repair and modulate the immune response. The secretion of SASP factors is not the only factor that reflects the unique function of senescent cells in the context of wound healing; their position at the edge of migrating cells is also significant. The separation of proliferating cells and migrating cells allows for the coordination of the wound closure and guidance of new blood vessel formation, which is facilitated by the process of senescence. The spatial distribution of senescent cells is of paramount importance, as their proximity to areas requiring ECM remodeling allows for targeted tissue restoration. Furthermore, senescent cells influence the plasticity of neighboring cells, supporting processes such as reprogramming and epithelial-mesenchymal transition through the release of factors such as IL-6. The presence of these cells is of particular importance during the initial stages following an injury, as evidenced by delayed wound healing in mouse models like p16-3MR, p16/p21 DKO, p21-ATTAC or pharmacological agents such as p21 inhibitors. While more experimental data is needed, it is possible that similarly spatially-restricted induction of senescence is important for healing of lung injury, traumatic brain injury, partial hepatectomy, among others.

### Evidence on the importance of spatial positioning of senescent cells in aging

Aging is a process of functional decline in biological systems over time. As an organism ages, damage accumulates, causing an increasing number of its subsystems to become dysregulated. Over time one or more of the sub-systems accumulates critical threshold of damage which leads to a critical failure of the affected sub system and consequently to the demise of the entire organism. It is currently thought that this progressive failure of biological subsystems manifests as age-associated diseases such as chronic kidney disease (CKD), diabetes, and non-alcoholic fatty liver disease (NAFLD), among others. Yet, it is not clear how organs and sub-systems transition from damage accumulation to the stage of the critical system failure. One line of thought suggests that cells that have acquired irreparable amount of damage throughout the aging process enter a cell cycle arrest and become senescent [80], [81]. While cellular senescence can occur under physiological conditions (as described in the previous two parts), a wide range of evidence suggests that in the context of aging, senescent cells are detrimental and may contribute to organ failure [82], [83], [84]. It is commonly believed that the formation of cellular senescence during the aging process is a random phenomenon due to the stochastic nature of cellular damage accumulation [85]. However, it has been shown that individuals and organs age at different rates [86]. Furthermore, it is known that there are differences in the rate of senescent cell accumulation across organs [87]. Therefore, it is reasonable to assume that certain regions of different organs may also age and accumulate senescent cells at varying rates. Another aspect of the spatial positioning of senescent cells in aged organs is their dynamic interaction with their surroundings through the SASP [88], [89], [90]. There is a substantial body of evidence suggesting that, especially in the context of aging, the SASP can induce senescence in neighboring cells, disrupt stem cell niches, impair tissue repair, and promote inflammation and fibrosis, which can lead to localized accumulation of senescent cells and tissue dysfunction [89], [90], [91], [92], [93]. The spatial organization of senescent cells is therefore crucial for understanding their influence on tissue function in the aging process. Below, we list examples of organs for which relevance of spatial positioning of aging-related senescent cells have been reported.

### Kidney

Kidney is an important organ which filters the blood from waste products, regulates electrolyte and fluid levels and produces certain hormones. The basic functional unit of the kidney is the nephron, which consists of the glomerulus (a cluster of capillaries) surrounded by the Bowman capsule, a cup-shaped structure, for blood filtration, and a tubular system (proximal tubule, loop of Henle, distal tubule, and collecting duct) that processes the filtrate into urine by reabsorbing water and essential solutes while secreting waste products. With aging the capacity of the kidney’s filtration rate declines. In particular, during aging the kidney undergoes nephrosclerosis (scarring of the glomeruli), interstitial fibrosis and tubular atrophy which can lead to CKD (defined as kidney damage or reduced glomerular filtration rate longer than 3 months) [94], [95], [96]. While increase of markers of senescence in kidney has been negatively correlated with human kidney transplant success as one of the first indications of the detrimental role of senescence in kidney function [97], below we list evidence on the relevance of spatial positioning of senescent cells in specific sub-structures of kidney: glomeruli and renal tubes.

The aging glomerulus undergoes several changes. One key change is the thickening of the glomerular basement membrane, a specialized extracellular matrix structure [95]. This membrane is located between fenestrated endothelial cells of the glomerular capillaries and the podocytes, that are wrapped around these capillaries. This structure serves as a selective filtration barrier in the kidney [98]. Another change is the expansion of the mesangial compartment [95]. This compartment is composed of the mesangial extracellular matrix and mesangial cells, which provide structural support to the glomerular capillary loops [98]. Additionally, mesangial cells play a role in regulating blood flow within the glomerulus. Thickening of the glomerular basement membrane in turn leads to glomerular enlargement and hyperfiltration (elevated glomerular filtration rate as a compensatory mechanism) [99]. Although this adaptation is initially beneficial, on the long-term it can cause renal injury [100]. Together with the ECM deposition this can lead to glomerulosclerosis and subsequently to a decline in glomerular filtration rate [95], [99].

In relation to the role of cellular senescence in this dysfunction, an age-dependent increase of p16 expression was observed in podocytes in kidney samples collected from human donors and mice [101]. Podocytes are highly specialized postmitotic epithelial cells in the kidney that play a crucial role in the filtration barrier that prevents the passage of large molecules such as proteins into the urine [102]. The senescent phenotype of these cells was further confirmed by immunoblot analysis of the isolated murine glomeruli at different age and in addition to upregulated p16 showed increases in p21, p53 and fibrogenesis-related SASP factors including TGF-β1, IGFBP3, and plasminogen activator inhibitor-1 (PAI-1) [101]. Additionally, an age-related increase of SA-β-gal signal in aged murine glomeruli was observed [101]. Another study has partially confirmed these findings using samples of rats and showing an age-related increase of p16-positive cells in the glomeruli but lack of the SA-β-gal signal [103]. The latter marker was found though in the renal tubules [103]. In contrast, another study investigating the presence of p16- and p21-positive cells in human samples with increasing age reported finding only a small number of p16-positive cells in the glomeruli [104]. This is in stark contrast to the study mentioned above, where a high abundance of p16-positive cells was observed in the glomeruli of human kidney donors [104].

In relation to potential drivers of podocyte senescence during aging, it was demonstrated that C/EBPα protects against podocyte senescence and aging-related kidney injury *in vivo* using a mouse model with podocyte-specific C/EBPα deletion [105]. Although C/EBPα is expressed in glomerular cells it has the highest expression in the podocytes [106] and podocytes are sensitive more than other kidney cells to reduce its expression upon stress [106], which could suggest that also aging affects particularly podocytes lowering C/EBPα expression. In aging mice, C/EBPα loss accelerated senescence, increased p16 expression, and worsened proteinuria, glomerulosclerosis, and tubulointerstitial fibrosis [105]. Morphological alterations, including podocyte foot process effacement, pathological flattening, and fusion of the normally interdigitating podocyte extensions, disrupt the slit diaphragm and impair the kidney's filtration barrier. This leads to protein leakage, progressive glomerular injury, and thickening of the glomerular basement membrane, which were more severe in C/EBPα-deficient mice. Mechanistically, the absence of C/EBPα disrupted autophagy via the AMPK/mTOR pathway, exacerbating tubular injury through albuminuria-induced epithelial–to-mesenchymal transition [105].

Surprisingly, another study identified glomerular endothelial cells, but not podocytes as the primary senescent cell type contributing to glomerulosclerosis, marked by increased expression of p16, p21, and SA-β-Gal [107]. There, senescent cells secrete PAI-1, which drives podocyte apoptosis and detachment through a pathological endothelial–podocyte cross-talk, leading to glomerular scarring. Senolytic approaches, including selective depletion of p16-positive cells using the INK-ATTAC transgenic model and endothelial-specific genetic inactivation of PAI-1, effectively reduced paracrine induction of podocyte apoptosis and ameliorated glomerulosclerosis in aged mice [107]. In summary, within the glomeruli of aging kidney primarily podocytes and endothelial cells become senescent, although there is some discrepancy in the observed markers across different studies. While it is currently unclear why cells of this specific anatomical location are becoming more frequently senescent than other kidney cells, podocytes being in the center of kidney’s filtration system are frequently exposed to toxins and other damaging agents, they have limited capacity for self-renewal and are more prone to DNA damage accumulation. Overall, while there is no direct evidence for it, induction of senescence in glomerular cell types might be initially protective (considering the alternative of apoptosis leading to leaky barrier due to cell loss and subsequently exposure of other cells to toxins), while in a long run leading to kidney dysfunction.

The second anatomical location in kidney with most frequently reported accumulation of senescent cells is the renal tubule, a part of the nephron, commencing just beyond the glomerulus. It plays an important role in filtering blood, reabsorbing essential substances, and excreting waste products. The renal tubule consists of the proximal convoluted tubule, the loop of Henle, the distal convoluted tubule, and the collecting duct [108]. A study on aging rats revealed a significant increase in p16-positive cells within the renal tubules and the surrounding interstitium [103]. Finally, this study found an increase of lipofuscin with increased age of rats, specifically in proximal, non-proximal and atrophic tubules (tubules that have shrunken and are dysfunctional due to chronic damage or injury) [103]. However, the highest increase of lipofuscin was observed in the proximal and atrophic tubules and the lowest in the non-proximal tubules [103]. To speculate on the reasons for high levels of senescence in proximal and damaged tubules, but not in non-proximal ones, we have to take into consideration their function. The renal proximal tubular epithelial cells (RPTECs) are required for the reabsorption of nutrients and solutes which is possible due to energy supply provided by abundant mitochondria [109]. The high mitochondrial activity in RPTECs, makes them produce high amount of ROS that induce oxidative DNA damage, cell cycle arrest and senescence mediated by p53/p21 and p16/pRB [109]. RPTECs often face these stresses due to exposure to toxins, hypoxia, and inflammatory mediators. Senescent proximal tubular cells have been shown to release several types of SASP factors such as pro-inflammatory cytokines (IL-1, IL-6), pro-fibrotic factors (TGF-β and MMPs) as well as other mediators such as monocyte chemoattractant protein-1 (MCP-1), which recruits immune cells and amplifies inflammation. These SASP factors perpetuate inflammation and renal fibrosis which drive the chronic drive the CKD progression [109].

In contrast, fewer senescent cells were identified in the distal part of the renal tubule [103]. The thick ascending limb cells (TAL) of the loop of Henle of the nephron is part of the distal renal tubules and is crucial for salt absorption, calcium and magnesium regulation, and acid-base balance, playing an essential role in urine concentration and dilution [110]. The discrepancy in the accumulation rate of senescent cells between the TAL and RPTEC might be multifaceted. Specifically, although the number of mitochondria is higher in the TAL compared to RPTEC, RPTECs have been shown to produce more ROS compared to the TAL [111]. Furthermore, RPTECs are responsible for the secretion and reabsorption of various endogenous and exogenous compounds which can occur in a transcellular or paracellular manner [112] as well as for metabolizing and excreting filtered xenobiotics [113]. In contrast, the distal parts of the nephron either do not or have limited reabsorption capacity [113]. Overall, it appears that spatially-distinct accumulation of senescent cells in proximal, but not in non-proximal might be due to their different quantities of mitochondria as well as differential exposure to toxins from the circulation.

While not directly connected to aging, it has been shown that a murine unilateral ischemia/reperfusion injury model shows an accumulation of senescent cells which leads to increased interstitial fibrosis as depicted by Masson, Fibronectin, Collagen I and α-SMA staining [114]. Furthermore, administration of dasatinib and quercetin (D+Q) reduced the number of senescent cells as well as fibrosis, which implies a direct role of senescent cells in the formation of kidney fibrosis and progression of CKD [114]. Moreover, the transfer of blood from old mice into young mice induced cellular senescence in the kidney (increased SA-β-gal staining) and markers of kidney damage like kidney injury molecule-1 (KIM-1, biomarkers for renal proximal tubular damage) [115].

**Figure 3:**
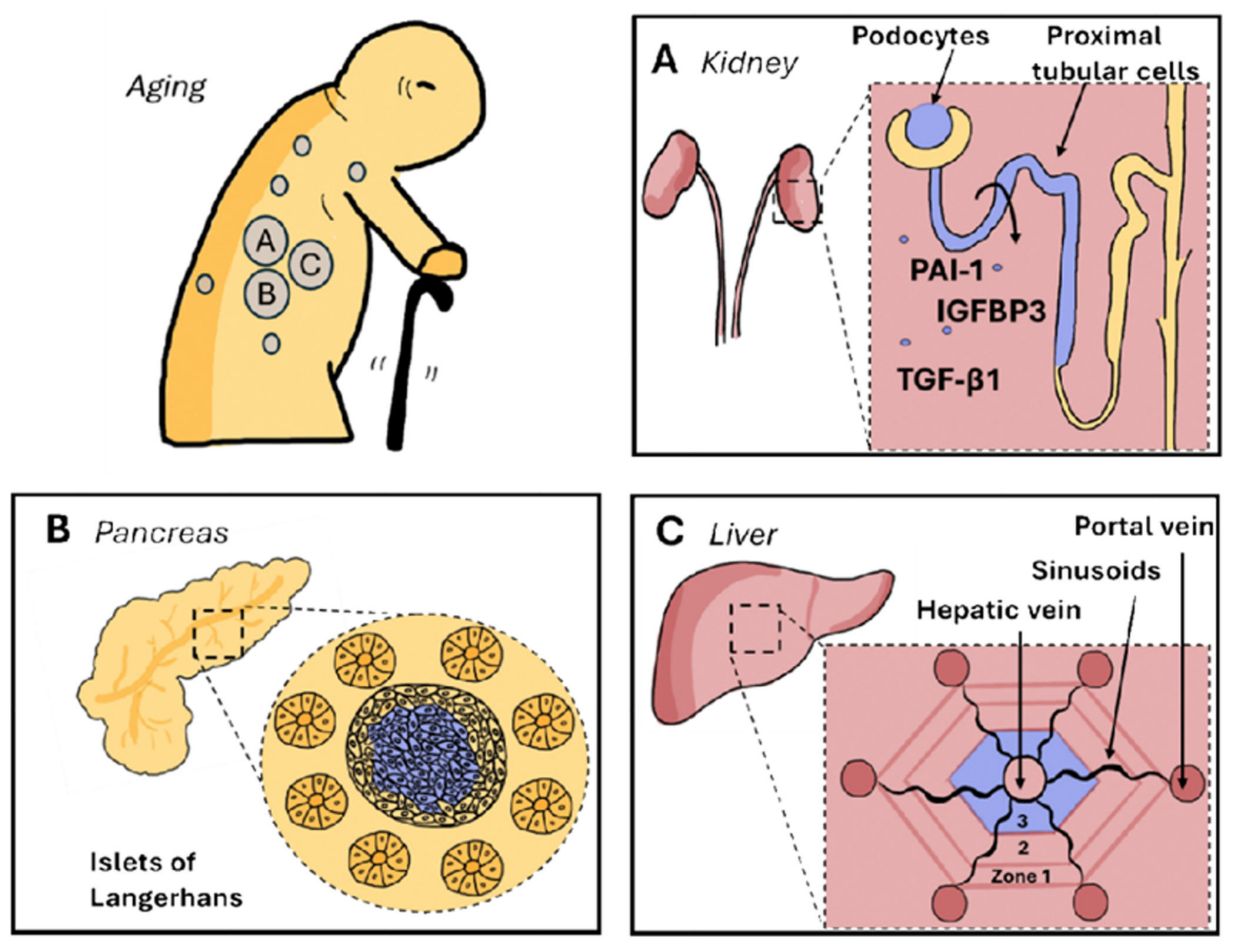
Spatial positioning of senescent cells during aging. (A) Senescent cells spatially accumulate in aging kidney in podocytes in the Bowmans capsule and proximal renal tubules. (B) In the aging pancreas senescent cells accumulate primarily in the beta cells of the islets of Langerhans. Senescent cells indicated in blue. (C) In the aging liver, the senescent cells accumulate primarily in zone 3 or pericentral hepatocytes

In summary, cellular senescence in kidney is highly localized around tubules (Fig. 3A) exposed to higher levels of toxins and harmful “waste products”. The spatial organization of senescent cells within these tubules might thus serve to resist apoptosis and function as a protective barrier. While this arrangement likely has positive short-term effects, the chronic persistence of these senescent cells ultimately leads to scarring and deterioration of kidney function.

### Pancreas

The pancreas regulates blood sugar levels through hormone secretion (endocrine function) and aids digestion by producing enzymes and bicarbonate (exocrine function). In the endocrine part of the pancreas alpha, beta, and pancreatic polypeptide cells (PP-cells) were shown to have increased markers of senescence with aging [116], [117], [118].

In the pancreas, cellular senescence markers have been observed to accumulate predominantly within the endocrine compartment, particularly in insulin-secreting beta cells of the islets of Langerhans [116]. Studies have demonstrated an age-associated increase in markers of senescence, such as p16 and p21, within these cells [116], [119]. Notably, p16 expression is already high in adult human beta cells but further increases with advancing age [116]. The functional implications of beta cell senescence are complex. Certain studies indicate that senescent beta cells may retain or even enhance insulin secretion capabilities, exhibiting higher levels of functional maturation, ability to effectively regulate blood glucose levels through precise insulin secretion, without displaying a strictly proinflammatory SASP [116], [120]. Conversely, other studies suggest that senescent beta cells experience a decline in identity markers and secrete proinflammatory factors, potentially contributing to local inflammation and impaired islet function [117]. Specifically, senescent beta cells in 7-to 8-month-old mice were identified through SA-β-gal staining and elevated p16 expression [117]. These senescent cells exhibited downregulation of genes essential for beta cell identity and increased secretion of pro-inflammatory cytokines, including TNF-α and CXCL1, compared to their non-senescent counterparts. In this study, the researchers induced beta cell senescence by creating insulin resistance through chronic administration of S961, an insulin receptor antagonist, and a high-fat diet [117]. Both interventions led to a rise in senescent beta cells, characterized by increased expression of SASP factors—such as IL-6, IL-1α, and TNF-α—and cell cycle arrest markers like p16 and p21. Notably, after discontinuing the insulin resistance-inducing treatments for two weeks, there was a reversal of aging markers and a partial reduction in SASP factors [117]. Additionally, beta cell functionality, assessed by the expression of genes like INS1, MAFA, and PDX1, improved post-recovery [117]. Another study is aligned with the latter study, that the beta cell function decreases with age and that different islets might have different aging rates [121]. Similarly, as to the previous study [117] acute administration of S961 induced senescence reversible after discontinuation [121].

In contrast to the endocrine part, the exocrine part seems to have a lower number of senescent cells, but a more pronounced increase during aging with an increase of both p16 and p21 [104]. In contrast, p16- and p21-positive senescent cells are already at high levels in young individuals and only p16-positive cells increase further with age (from 15% up to 35% of cells) [104]. The difference between senescent cell accumulation between the exocrine and endocrine part might be attributed to their regenerative capacity – possibility to replace damaged cells. Namely, the exocrine part is able to fully regenerate from acutely induced pancreatitis in rodents [122]. Conversely, the regenerative capacity of the endocrine part declines sharply after a pancreatectomy when reaching adulthood [122]. Furthermore, beta-cell regeneration was not observed after administering a beta cell specific ablative drug [122]. Nevertheless, beta cell hyperplasia has been observed under certain conditions such as pregnancy, obesity, and insulin resistance [122]. This phenotype in the pancreas would be similar as we have already discussed for the senescence of podocytes, which might be susceptible to senescence due to post-mitotic state.

The spatial confinement of senescent cells to the endocrine part of the pancreas, especially beta cells, may be attributed to the high metabolic demands and continuous exposure to glucose and insulin signaling inherent to these cells. Chronic hyperinsulinemia has been shown to promote cellular senescence in various cell types, such as adipocytes, neurons and hepatocytes, by activating the p53/p21 signaling pathway through prolonged insulin receptor signaling, particularly via the PI3K/AKT/mTOR pathway [123]. It is thus possible that this persistent metabolic activity renders beta cells susceptible to stress-induced senescence.

In summary, senescent cells in the pancreas are primarily localized within the endocrine islets (Fig. 3B), especially beta cells. Their presence influences tissue function, potentially affecting insulin secretion and contributing to inflammatory processes. The spatial distribution of these senescent cells is likely a consequence of the intrinsic metabolic demands and signaling exposures characteristic of pancreatic beta cells.

### Liver

The liver is a central organ maintaining physiological homeostasis through its highly structured hexagonal anatomical units called liver lobules. Blood flows inward from the oxygen-rich portal tracts to the central vein, creating gradients that establish non-uniform distribution of liver functions along the lobule's radial axis, a phenomenon known as “liver zonation”. Hepatocytes, organized in radial hepatic plates, interact with endothelial cells, hepatic stellate cells, and Kupffer cells to mediate nutrient uptake, hormone sensing, detoxification, and metabolic processes. Liver zonation optimizes metabolic processes by spatially distributing tasks along the lobule's radial axis, matching oxygen availability and functional demands. Periportal hepatocytes (zone 1), exposed to higher oxygen levels, are involved in energy-intensive processes such as gluconeogenesis, urea synthesis and cholesterol biosynthesis. In contrast, pericentral hepatocytes (zone 3), located in oxygen-deprived areas, exhibit higher metabolic activity for less oxygen-dependent processes such as xenobiotic metabolism, bile acid biosynthesis, glycolysis, and the recycling of amino acids like glutamate and glutamine [124]. Low oxygen levels and high detoxification activity of this region is reflected with higher cytochrome P450 levels, enzymes that convert drugs into water-soluble products for easier elimination [125].

Markers of cellular senescence have been observed accumulating in hepatocytes with aging, particularly in the pericentral hepatocytes (zone 3) [126]. While it has not been directly proven, the breakdown of toxins and the activity of P450 enzymes in this region is associated with high ROS production causing oxidative damage [127], [128], which might lead to higher senescent cell load in this region. Oxidative stress intensifies with age, leading to the accumulation of senescent cells, as evidenced by markers such as DNA damage foci and SA-β-gal staining [126]. Notably, DNA damage is spatially correlated with markers of oxidative stress, such as 4-hydroxynonenal, suggesting that oxidative stress-induced DNA damage drives hepatocyte senescence [126].

The spatial distribution of senescent cells aligns with the initial formation of steatosis in NAFLD, which predominantly occurs in zone 3 of the liver in adults [129]. Furthermore, fat deposition around the central vein in aging was shown also by Oil-red O and PLIN2 immunohistochemical stainings [130]. Notably, senescent cells have been implicated in promoting fat deposition within the liver [131]. This study demonstrated that in murine models of NAFLD and in aged mice the targeted elimination of senescent cells, either through suicide gene-mediated ablation of p16-expressing cells in INK-ATTAC mice or via treatment with D+Q, significantly reduces hepatic steatosis [131], although it was not shown whether this effect is more prominent around the central vein/zone 3. Similarly, hepatocyte ballooning, a pathological enlargement and cytoplasmic clearing caused by cytoskeletal disruption, intracellular stress, and lipid accumulation, serving as a hallmark of cellular injury in liver diseases like non-alcoholic steatohepatitis (NASH), is shown to initially occur in zone 3 [129]. Overall, the spatial distribution of senescent cells in zone 3 (Fig. 3C) appears to serve a dual role: initially resisting apoptosis and acting as a barrier to further damage, but eventually contributing to age-related pathologies, such as hepatic steatosis [132]. This progression might underscore the paradoxical nature of senescence, with short-term protective effects giving way to chronic dysfunction and tissue deterioration with age. In summary, while there is evidence that certain parts of the liver, especially those providing a barrier to toxins, are more sensitive to the accumulation of senescent cells in a wide range of pathological conditions, detailed data on the spatiotemporal distribution of senescent cells and its pathophysiological consequences remain to be fully elucidated.

To summarize the relationship between the spatial positioning of senescent cells and the aging process, the currently available data suggests that the distribution of most senescent cells is predominantly non-random. These cells tend to cluster around certain structures, such as the central vein of the liver or kidney tubules, and this positioning is not dependent on cell type. In the kidney, for instance, the proximal tubules, which are characterized by high metabolic activity and detoxification processes, appear to be particularly prone to cellular senescence. Similarly, in the liver, zone 3, known for its elevated metabolic activity and detoxification functions, harbors a higher concentration of senescent cells. In the pancreas, beta cells exhibit a greater propensity for senescence, likely due to the heavy protein production load they endure and higher sensitivity to oxidative stress. In general, the formation of senescent cells in these specific zones may represent a temporary response to toxins or high metabolic activity, functioning as a “shock absorber” or barrier against initial insults. The induction of the senescent state protects cells from undergoing apoptosis, allowing them to maintain tissue integrity under adverse conditions. In contrast, without the activation of cellular senescence, cells in these environments would be more susceptible to apoptosis and necrosis, which could lead to tissue disruption and compromised organ function. This protective role of senescence is supported by experimental evidence. For example, short-term exposure to toxins from heterochronic blood exchange from aged mice introduced into young mice results in markers of kidney damage and cellular senescence in the young mice shortly after administration [115]. While this acute activation of senescence can be beneficial in mitigating immediate damage, prolonged activation of the senescent phenotype leads to impairments and disruptions in the local tissue environment. This highlights the dual nature of cellular senescence: a protective mechanism in the short term, but a potential driver of pathology if sustained over time. We speculate that especially tissues on the crucial interface between the body and the environment such as lung or endometrium might be showing phenotypes during aging consistent with the examples described above.

### How senescent cells exert their spatial influence: barriers and conductors

In the context of complex biological processes such as tissue development, wound healing, and regeneration, not only are distinct cellular phenotypes important, but their strategic spatial positioning is also crucial. Notably, senescent cells, which enter a state of growth arrest in response to various stressors, appear to be strategically positioned, occurring in diverse physiological and pathophysiological settings [10], [11], [14], [48]. Here, we posit that senescent cells require specific spatial positioning to execute their functions; this positioning is beneficial in the context of physiology, but in conditions such as aging and pathologies, it can be detrimental, especially long-term. Furthermore, in relation to the physiology of cellular senescence, we propose that the spatially-relevant functions of senescent cells can be divided into "barrier" and "conductor" functions.

It appears that senescent cells serve as a "barrier" by occupying pivotal locations between disparate cell layers and tissues. Such cells are located at key points, including the vicinity of the wound edge [48], [50], [68], [133]. The induction of cell cycle arrest in keratinocytes at the edge of the migrating front during wound closure represents a type of "barrier" between healthy and damaged tissue [50], [133]. The migrating front during re-epithelialization, as well as the tip cells of growing blood vessels, exhibit characteristics of cellular senescence, thereby clearly delineating non-proliferating leader cells from proliferating stalker cells [73]. During limb development, the position at the edge of the limb can be conceptualized as a barrier that spatially confines the apoptosis necessary for the separation of fingers [10]. The separation between mother and fetus is another illustration of the role of senescence as a "barrier". The distinctive morphology of senescent cells, including their enlarged and flattened appearance and cell cycle arrest, is crucial for the establishment and maintenance of a functional placental STB [31], [32]. In the context of aging, senescent cells appear at the interface between circulation and tissue interstitium. It appears that both kidney and liver acquire senescent cells in regions key for detoxification, such as renal tubules for the kidney and zone 3 for the liver [103], [109], [126].

The transmission of signals to neighbouring cells represents a hallmark of senescence and can be conceptualized as their "conductor" function. This function is performed by the SASP, that can profoundly influence the tissue microenvironment [55], [134], [135]. The localization of these cells within tissues is not arbitrary but rather highly strategic, significantly affecting their functional impact on surrounding cells and the extracellular matrix. Senescent cells appear at the edge of developing limbs and influence the adjacent tissue through the secretion of signalling molecules, ensuring proper patterning and growth [10]. During wound healing, the secretion of specific factors, such as PDGF-AA and EPGN, is essential for proper wound closure [48], [50]. Additionally, SASP can influence immune cell responses and attract immune cells to the wound site, which is a crucial step in healing [49], [64]. As a consequence of the aging process, the increase in the number of senescent cells becomes chronic; the conductor function contributes to tissue inflammation via SASP and results in the pathologies observed during the aging process. The other facet of how this function gets downregulated during aging is that it becomes depleted, as observed in the pancreas [117], [121]. With ample examples of how spatial induction of senescent cells affects physiology and pathology, we believe that categorizing these cells into 'barriers' and 'conductors' might facilitate mapping the roles of cellular senescence *in situ*.

## Conclusions

We are now experiencing the era of the "spatial revolution" in biology, with a plethora of spatial omics techniques emerging and/or becoming commonly used. The field of cellular senescence research is yet to fully catch up and is currently challenged by how to define senescent cells sufficiently accurately to enable reliable detection via omics [136]. While there has already been progress in providing criteria for reliable detection of senescent cells *in vivo* and *in situ* [137], these are yet to be applied in spatial omics applications. To aid efforts of detailed senescence mapping *in situ*, we review here what is known about specific anatomical locations known to harbor senescent cells under physiological and pathological conditions. Overall, we conclude that the spatial restriction of senescent cells ensures their confinement to areas where they are most needed for precise morphogenetic, tissue damage, and healing events, thereby facilitating optimal tissue function and repair. To simplify the variety of contexts and physiological features of the spatial impact of senescence, we categorize them into "barriers" and "conductors," which might provide an easier entry point for newcomers to the field of cellular senescence and help experts in senescence research navigate through the spatially-relevant phenotypic characteristics of cellular senescence.
